# A numerical analysis of buckle cable force of concrete arch bridge based on stress balance method

**DOI:** 10.1038/s41598-022-15755-w

**Published:** 2022-07-21

**Authors:** Zengwu Liu, Shuixing Zhou, Kairan Zou, Yinghao Qu

**Affiliations:** School of Civil Engineering, Chongqing Jiaotong University, Chongqing, 400074 China

**Keywords:** Engineering, Civil engineering

## Abstract

It is difficult to calculate the buckle cable force for the cantilever casting concrete arch bridge. Relying on the 180 m-span Jiming Sansheng Bridge, this paper put forward one method to calculate the initial buckle cable force based on the stress balance method. Firstly, the stress balance equation considering only tensile stress was derived for the first time, and the feasible region of the initial cable force was calculated by the allowable tensile stress of the arch rib, which improved the original stress balance method. Then, using the influence matrix, the initial buckle cable force was optimized by reducing the allowable tensile stress of concrete in stages, and finally the optimal initial cable force was obtained. The practical engineering results show that it is feasible to calculate the initial buckle force. The maximum tensile stress of concrete arch during the cantilever casting process is 1.52 MPa, meeting the specification requirements. The deviation between the calculated and measured stress is less than 12%. The calculated cable force agrees with the measured cable force, and the deviation is less than 2%. The initial cable force is only tensioned once, improving work efficiency. The method and experience of this paper can provide a reference for the arch bridge constructed by cantilever casting.

## Introduction

In the 1950s, the arch bridge began to be built by cantilever construction technology. In the 1960s, Former Yugoslavia completely adopted the cantilever casting method to successfully build two large-span arch bridges. In the 1970s and 1980s, South Africa, Europe and other countries successively used the cantilever casting method to build a number of large-span arch bridges. For example, Croatia used the cantilever casting method to build the Maslenica Bridge and Krka Bridge with spans of 200 and 204 m respectively in 1997 and 2005. In the Republic of South Africa, the Bloukrans bridge (272 m) was built by cantilever casting in 1983. In recent years, many large span arch bridges with novel structure and advanced technology have been built by the cantilever casting method abroad, which has created span records of similar arch bridges in China and even in the world. The cantilever casting construction technology of arch bridge has been rapidly developed abroad and has achieved good economic and social benefits.

Many scholars^1,2^ have studied the initial cable force of cable structure bridges. For the special-shaped arch bridge, the reasonable design method of suspender was proposed^3^. Genetic algorithm^4^ was used to optimize the structure of the reinforced concrete arch bridge. The mechanical behavior of the arch bridge was studied by the finite element method and verified by experiment^5^. Dai et al.^6^ proposed the cable force calculation method of the arch bridge construction process, which provided guidance for the cable force calculation of similar bridges. The cable forces^7,8^ were calculated by constraining the deformation of cable-stayed bridges. The initial cable forces^9^ of the cable structure bridge were determined by using entropy theory. The cable force^10^ was determined by the reasonable moment distribution of the girder. Taking a 150 m-span concrete arch bridge as an example, Li et al.^11^ studied the scaled model test of the arch bridge, and used the stress balance method to calculate the force of the buckle cable. Qi et al.^12^ used ANSYS first-order optimization method to study the cable force adjustment method under the maximum cantilever state during the cantilever casting construction of the arch bridge. Zhou et al.^13^ took the maximum tensile stress square sum of the arch rib section as the goal and the initial buckle force as a variable to optimize the initial buckle force of the cantilever casting arch bridge. According to the cantilever casting process of arch rib, Hu et al.^14^ put forward the zero moment method to calculate the initial cable force, relying on the 180 m Mati River Bridge. The results showed that the zero moment method could effectively calculate the initial buckle force. Granata et al.^15^ put forward one method to calculate the cable force with the geometric shape of the arch rib as the goal, and an example was designed to verify the calculation method. The results showed that the presented method could improve the calculation efficiency of the initial buckle cable force. However, the calculation methods of initial buckle force of concrete arch bridge with cantilever casting construction method were still very few. In addition, in the existing literature, the cable was not tensioned at one time, but was tensioned twice, or the cable force was readjusted when the arch rib was at the maximum cantilever.

In the aspect of cable simulation, Ernst et al.^16^ proposed the equivalent elastic modulus method of cable structure to accurately consider the mechanical behavior of cables. Fleming et al.^17^ proposed to use a truss element to simulate the cable, and use the equivalent elastic modulus method to consider the nonlinear effect of the cable. Liang^18^ and Xia et al.^19^ pointed out that the deformation of cable is the same as that of catenary, and proposed using the catenary element to simulate cable. In this paper, the cable was simulated by truss element and modified by the Ernst formula.

In the process of arch rib construction, if the buckle and anchor cables are only tensioned once, the construction time will be saved. Moreover, if the cable is tensioned repeatedly, the clip of the anchor cable will damage the cable and increase the risk in the process of arch rib construction.

For the cantilever casting concrete arch bridge, this paper put forward one method to calculate the initial buckle cable force. Firstly, the stress balance equation considering only tensile stress was derived for the first time, and the feasible region of the initial cable force was calculated by the allowable tensile stress of the arch rib. Secondly, using the influence matrix, the initial buckle cable force was optimized by reducing the allowable tensile stress of concrete in stages. Finally, the method was applied to calculate the initial cable force of Jiming Sansheng Bridge during cantilever construction, and the buckle and anchor cables were only tensioned once in the process of arch rib construction.

## Introduction of cantilever casting process of arch rib

**Figure 1:**
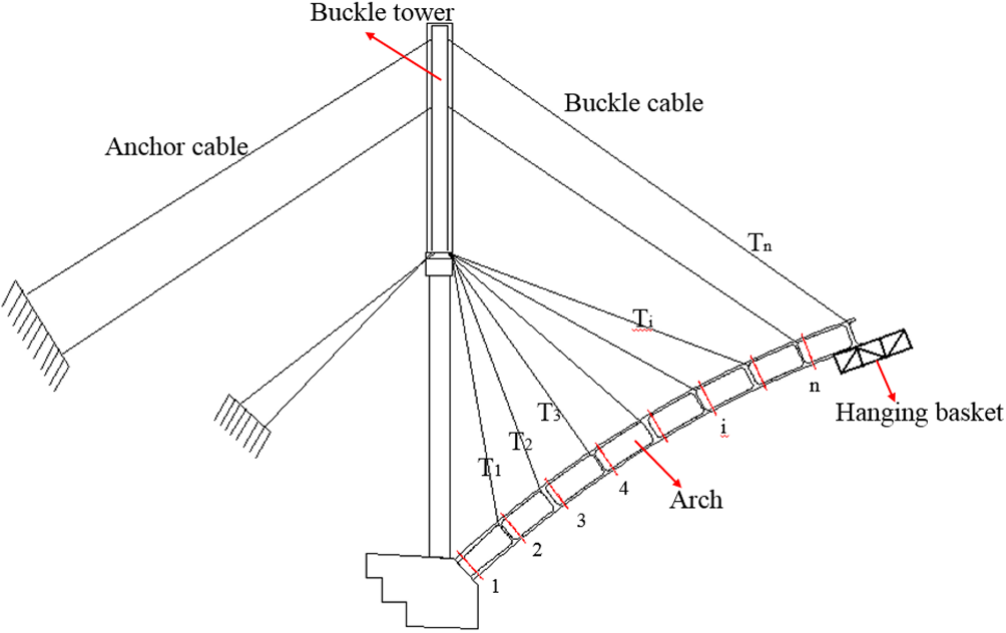
Cantilever casting arch rib construction system.

As shown in Fig. 1, the cantilever casting arch rib construction system is mainly composed of buckle tower, buckle and anchor cables, a concrete pier, and a hanging basket. The arch rib is poured with buckle cable, anchor cable and hanging basket cantilever. After the strength of segment *i* concrete reaches 85%, the No. *i* buckle cable is tensioned. Then, the hanging basket is moved to the (*i* + 1) section, and the concrete of the (*i* + 1) section is poured. The tension ends of the cable and anchor cable are on the tower. To reduce the deviation of the tower, the cable and anchor cable are tensioned at the same time. The hanging basket is anchored on the upper of the arch rib by the suspender, and it can move along the arch rib.

## Calculation method and process of initial buckle cable force

The initial feasible region of buckle cable force is calculated by the stress balance method. In the process of cantilever casting of arch rib, arch rib stress is mainly caused by the gravity of arch rib, temporary load (mainly hanging basket load) and buckle cable force. To ensure the construction quality and safety of the arch rib in the construction process, the tensile and compressive stress of the arch rib section must satisfy the requirements of allowable stress. Assuming that the cantilevered arch rib has *n* segments and *n* buckle cables as shown in Fig. 1. There are corresponding n key sections, because the internal force of the section at the cable support of the arch rib segment is the largest.

The upper edge stress of the key section of the arch rib is vector $\left\{ {\sigma^{t} } \right\}$, and the lower edge stress is vector $\left\{ {\sigma^{b} } \right\}$. See Eqs. (1) and (2). The stress of the key section of the arch rib can be obtained by using Eqs. (1) and (2).

1 $$ \left\{ {\sigma^{t} } \right\} = \left\{ {\sigma_{g}^{t} } \right\} + A\left\{ T \right\} + \left\{ {\sigma_{P}^{t} } \right\} $$

2 $$ \left\{ {\sigma^{b} } \right\} = \left\{ {\sigma_{g}^{b} } \right\} + B\left\{ T \right\} + \left\{ {\sigma_{P}^{b} } \right\}, $$

where $\left\{ {\sigma^{t} } \right\}$ represents the stress vector of the upper edge of the key sections, that is, $\left\{ {\sigma^{t} } \right\} = \left( {\sigma_{1}^{t} ,\sigma_{2}^{t} ,\sigma_{3}^{t} , \ldots ,\sigma_{n}^{t} } \right)_{1 \times n}^{{\text{T}}}$. $\left\{ {\sigma^{b} } \right\}$ represents the stress vector of the lower edge of the key sections, that is, $\left\{ {\sigma^{b} } \right\} = \left( {\sigma_{1}^{b} ,\sigma_{2}^{b} ,\sigma_{3}^{b} , \ldots ,\sigma_{n}^{b} } \right)_{1 \times n}^{{\text{T}}}$. $\left\{ {\sigma_{g}^{t} } \right\}$ represents the stress vector at the upper edge of the key sections caused by the weight of the arch rib, that is, $\left\{ {\sigma_{g}^{t} } \right\} = \left( {\sigma_{g1}^{t} ,\sigma_{g2}^{t} ,\sigma_{g3}^{t} , \ldots ,\sigma_{gn}^{t} } \right)_{1 \times n}^{{\text{T}}}$. $\left\{ {\sigma_{g}^{b} } \right\}$ represents the stress vector at the lower edge of the key sections caused by the weight of the arch rib, that is, $\left\{ {\sigma_{g}^{b} } \right\} = \left( {\sigma_{g1}^{b} ,\sigma_{g2}^{b} ,\sigma_{g3}^{b} , \ldots ,\sigma_{gn}^{b} } \right)_{1 \times n}^{{\text{T}}}$. $\left\{ {\sigma_{p}^{t} } \right\}$ represents the stress vector at the upper edge of the key sections caused by the temporary load, that is, $\left\{ {\sigma_{p}^{t} } \right\} = \left( {\sigma_{p1}^{t} ,\sigma_{p2}^{t} ,\sigma_{p3}^{t} , \ldots ,\sigma_{pn}^{t} } \right)_{1 \times n}^{{\text{T}}}$. $\left\{ {\sigma_{p}^{b} } \right\}$ represents the stress vector at the lower edge of the key sections caused by the temporary load, that is, $\left\{ {\sigma_{p}^{t} } \right\} = \left( {\sigma_{p1}^{t} ,\sigma_{p2}^{t} ,\sigma_{p3}^{t} , \ldots ,\sigma_{pn}^{t} } \right)_{1 \times n}^{{\text{T}}}$. $\left\{ T \right\}$ is the buckle forces vector, that is, $\left\{ T \right\} = \left( {T_{1} ,T_{2} ,T_{3} , \ldots ,T_{n} } \right)_{1 \times n}^{{\text{T}}}$.

$A$ represents the influence matrix of the buckle force on the upper edge stress of the key sections.

$B$ represents the influence matrix of the buckle force on the lower edge stress of the key sections.

For example, $a_{ij}$ represents the stress produced by a unit force acting on the upper edge of the key section *i* of buckle cable *j*.

In the cantilever casting construction stage, self-weight load and temporary load always produce tensile stress on the upper edge of arch rib key sections, and compressive stress on the lower edge. The buckle force produces compressive stress on the upper edge of the arch rib key sections and tensile stress on the lower edge of the arch rib key sections. The arch rib supported by the cable is equivalent to the curved cantilever beam. The compressive stress of arch rib during construction is less than that of arch rib after closure, and the concrete has a strong compressive capacity, but the tensile capacity is weak. However, if the tensile stress of arch rib key sections exceeds the allowable tensile stress, the arch rib will crack. The allowable tensile stress of arch rib key sections could be determined according to the specification^20^.

In this study, the allowable tensile stress of arch rib key sections is expressed as $\left\{ {f^{t} } \right\}$.When casting arch rib concrete, it is necessary to pay attention to the tensile stress at the upper edge of arch rib key sections. $\left\{ {f^{t} } \right\}$ was substituted into Eq. (1) to get Eq. (3).

3 $$ \left\{ {\sigma^{t} } \right\} = \left\{ {\sigma_{g}^{t} } \right\} + A\left\{ T \right\} + \left\{ {\sigma_{P}^{t} } \right\} < \left\{ {f^{t} } \right\} $$

When tensioning the buckle cable force, it is necessary to pay attention to the tensile stress at the lower edge of arch rib key sections. $\left\{ {f^{t} } \right\}$ was substituted into Eq. (2) to get Eq. (4) . Equations (3) and (4) can be used to ensure that the tensile stress of the key section of the arch rib is less than the allowable tensile stress.

4 $$ \left\{ {\sigma^{b} } \right\} = \left\{ {\sigma_{g}^{b} } \right\} + B\left\{ T \right\} + \left\{ {\sigma_{P}^{b} } \right\} < \left\{ {f^{t} } \right\} $$

In Eqs. ( 3 ) and ( 4 ), tensile stress is positive and compressive stress is negative. Eqs. ( 3 ) and ( 4 ) can be converted into Eqs. (5) and (6).

5 $$ A\left\{ T \right\} < \left\{ {f^{t} } \right\} - \left\{ {\sigma_{g}^{t} } \right\} - \left\{ {\sigma_{P}^{t} } \right\} $$

6 $$ B\left\{ T \right\} < \left\{ {f^{t} } \right\} - \left\{ {\sigma_{g}^{b} } \right\} - \left\{ {\sigma_{P}^{b} } \right\} $$

Equations (5) and (6) were converted, and Eqs. (7) and (8) can be obtained.

7 $$ \left\{ \begin{gathered} a_{11} T_{1} + a_{12} T_{2} + a_{13} T_{3} \cdots + a_{1n} T_{n} < f_{{}}^{t} - \sigma_{g1}^{t} - \sigma_{P1}^{t} \hfill \\ a_{21} T_{1} + a_{22} T_{2} + a_{23} T_{3} \cdots + a_{2n} T_{n} < f_{{}}^{t} - \sigma_{g2}^{t} - \sigma_{P2}^{t} \hfill \\ \begin{array}{*{20}c} {\begin{array}{*{20}c} {\begin{array}{*{20}c} {\begin{array}{*{20}c} {} & {} & {} \\ \end{array} } & {} & {} \\ \end{array} } & {} & {} \\ \end{array} } & \vdots & {} \\ \end{array} \hfill \\ a_{n1} T_{1} + a_{n2} T_{2} + a_{n3} T_{3} \cdots + a_{nn} T_{n} < f_{{}}^{t} - \sigma_{gn}^{t} - \sigma_{Pn}^{t} \hfill \\ \end{gathered} \right. $$

8 $$ \left\{ \begin{gathered} b_{11} T_{1} + b_{12} T_{2} + b_{13} T_{3} \cdots + b_{1n} T_{n} < f_{{}}^{t} - \sigma_{g1}^{b} - \sigma_{P1}^{b} \hfill \\ b_{21} T_{1} + b_{22} T_{2} + b_{23} T_{3} \cdots + b_{2n} T_{n} < f_{{}}^{t} - \sigma_{g2}^{b} - \sigma_{P2}^{b} \hfill \\ \begin{array}{*{20}c} {\begin{array}{*{20}c} {\begin{array}{*{20}c} {\begin{array}{*{20}c} {} & {} & {} \\ \end{array} } & {} & {} \\ \end{array} } & {} & {} \\ \end{array} } & \vdots & {} \\ \end{array} \hfill \\ b_{n1} T_{1} + b_{n2} T_{2} + b_{n3} T_{3} \cdots + b_{nn} T_{n} < f_{{}}^{t} - \sigma_{gn}^{b} - \sigma_{Pn}^{b} \hfill \\ \end{gathered} \right. $$

Since the stress influence matrices $A$ and $B$ are upper triangular matrices, the feasible region of the buckle cable force can be obtained by Eqs. (7) and (8). The minimum cable force $T_{n}^{\min }$ is shown in Eq. ( 9 ), and the maximum cable force $T_{n}^{\max }$ is shown in Eq. (10).

9 $$ T_{n}^{\min } = \max \left[ {\left( {f_{{}}^{t} - \sigma_{gn}^{t} - \sigma_{pn}^{t} } \right)/a_{nn} ,0} \right] $$

10 $$ T_{n}^{\max } = \min \left[ {\left( {f_{{}}^{t} - \sigma_{gn}^{b} - \sigma_{pn}^{b} } \right)/{\text{b}}_{nn} } \right], $$

where $\sigma_{gn}^{t}$ represents the upper edge stress of the key section $n$ caused by the weight of the arch rib.$\sigma_{gn}^{b}$ represents the lower edge stress of the key section $n$ caused by the weight of the arch rib. $\sigma_{pn}^{t}$ represents the upper edge stress of the key section $n$ caused by the temporary load.$\sigma_{pn}^{b}$ represents the lower edge stress of the key section $n$ caused by the temporary load. $f_{{}}^{t}$ is the allowable tensile stress of the upper and lower edges of the key section $n$. $a_{nn}$ and $b_{nn}$ represent the compressive stress on the upper edge and tensile stress on the lower edge of the key section $n$ when the cable $n$ acts on a unit force, respectively.

The feasible region $T_{n}^{{}} \in \left[ {T_{n}^{\min } ,T_{n}^{\max } } \right]$ of the initial force $T_{n}^{{}}$ of buckle cable $n$ can be calculated by Eqs. (9) and (10), and the tensile stress of the arch rib can meet the requirements.

The feasible region of initial force $T_{n - 1}^{{}}$ can be calculated by substituting the initial force $T_{n}^{{}}$ of buckle cable $n$ into Eqs. (7) and (8).

Moreover, the feasible region of the initial force $T_{i}^{{}}$ of cable $i$ is $T_{i}^{{}} \in \left[ {T_{i}^{\min } ,T_{i}^{\max } } \right]$. The minimum cable force $T_{i}^{\min }$ is shown in Eq. (11), and the maximum cable force $T_{i}^{\max }$ is shown in Eq. (12).

11 $$ T_{i}^{\min } = \max \left[ {\left( {f_{{}}^{t} - \sigma_{gi}^{t} - \sigma_{pi}^{t} - \sum\limits_{j = i + 1}^{n} {a_{ij} T_{j}^{\min } } } \right)/a_{ii} ,0} \right],\;\;\;(i = 1,2, \ldots n - 1) $$

12 $$ T_{i}^{\max } = \min \left[ {\left( {f^{t} - \sigma_{gi}^{b} - \sigma_{pi}^{b} - \sum\limits_{j = i + 1}^{n} {b_{ij} T_{j}^{\max } } } \right)/{\text{b}}_{ii} } \right],\;\,\,(i = 1,2, \ldots n - 1), $$

where $\sigma_{gi}^{t}$ represents the upper edge stress of the key section $i$ generated by the weight of the arch rib.$\sigma_{gi}^{b}$ represents the lower edge stress of the key section $i$ generated by the weight of the arch rib. $\sigma_{pi}^{t}$ represents the upper edge stress of the key section $i$ generated by the temporary load. $\sigma_{pi}^{b}$ represents the lower edge stress of the key section $i$ generated by the temporary load. $a_{ij}$ and $b_{ij}$ represent the compressive stress on the upper edge and tensile stress on the lower edge of the key section $i$ when the cable $j$ acts on a unit force, respectively.

According to Eqs. (11) and (12), the feasible region of all cable forces can be calculated. The cable force in the feasible region can ensure that the tensile stress of the arch rib meets the requirements. However, a group of optimal cable forces is needed in the actual cantilever casting construction process.

Suppose a group of buckle cable forces $\left\{ {T^{1} } \right\}$ is recorded as $\left\{ {T^{1} } \right\} = (T_{1}^{1} ,T_{2}^{1} ,T_{3}^{1} \ldots T_{n}^{1} )_{1 \times n}^{{\text{T}}}$,$T_{i}^{1} \in \left[ {T_{i}^{\min } ,T_{i}^{\max } } \right]$,$i = 1,2, \ldots ,n$. $\left\{ {T^{1} } \right\}$ is substituted into Eqs. (1) and (2), the upper edge stress $\left\{ {\sigma^{t1} } \right\}$ and lower edge stress $\left\{ {\sigma^{b1} } \right\}$ of key sections of the arch rib are determined respectively. The upper edge stress and the lower edge stress satisfy Eqs. (3) and (4), that is, $\left\{ {\sigma^{t1} } \right\}$ and $\left\{ {\sigma^{b1} } \right\}$ are both less than $\left\{ {f^{t} } \right\}$. But if the value $\left\{ {f^{t} } \right\}$ is reduced, Eqs. (3) and (4) may not be satisfied.

This paper proposes a method to reduce the allowable tensile stress $f^{t}$ of concrete to optimize the initial buckle force, and the influence matrix is used in the optimization process. The optimization process of the initial buckle force is as follows, where the reduced allowable tensile stress of concrete is defined as $f{{^\prime}^{t}}$.

Step 1: Substitute $\left\{ {T^{1} } \right\}$ into Eqs. (1) and (2), the upper edge stress $\left\{ {\sigma^{t1} } \right\}$ and lower edge stress $\left\{ {\sigma^{b1} } \right\}$ of the key section of the arch rib are calculated respectively ;

Step 2: Check whether $\left\{ {\sigma^{t1} } \right\}$, $\left\{ {\sigma^{b1} } \right\}$ and $f^{^{\prime}t}$ satisfy Eqs. (3) and (4). If they are satisfied, continue to reduce the value $f^{^{\prime}t}$. Otherwise, the cable force $\left\{ {T^{1} } \right\}$ should be adjusted;

Step 3: Combine Eqs. (1) and (2), the cable force adjustment vectors $\left\{ {\Delta T^{t} } \right\}$ and $\left\{ {\Delta T^{b} } \right\}$ can be calculated by Eq. (13):

13 $$ \left\{ \begin{gathered} \left\{ {\Delta T^{t} } \right\} = A^{ - 1} (\left\{ {f^{^{\prime}t} } \right\} - \left\{ {\sigma^{t1} } \right\}) \hfill \\ \left\{ {\Delta T^{b} } \right\} = B^{ - 1} (\left\{ {f^{^{\prime}t} } \right\} - \left\{ {\sigma^{b1} } \right\}) \hfill \\ \end{gathered} \right. $$

Step 4: Select the maximum value of $\left\{ {\Delta T^{t} } \right\}$ and $\left\{ {\Delta T^{b} } \right\}$ as $\left\{ {\Delta T} \right\}$. Then, $\left\{ {T^{1} + \Delta T} \right\}$ is substituted into Eqs. (1) and (2) to re-calculate key section stress of arch rib, and whether it matches the Eqs. (3) and (4) are checked. If they are satisfied, continue to decrease $f^{^{\prime}t}$ and repeat steps one to four.

**Figure 2:**
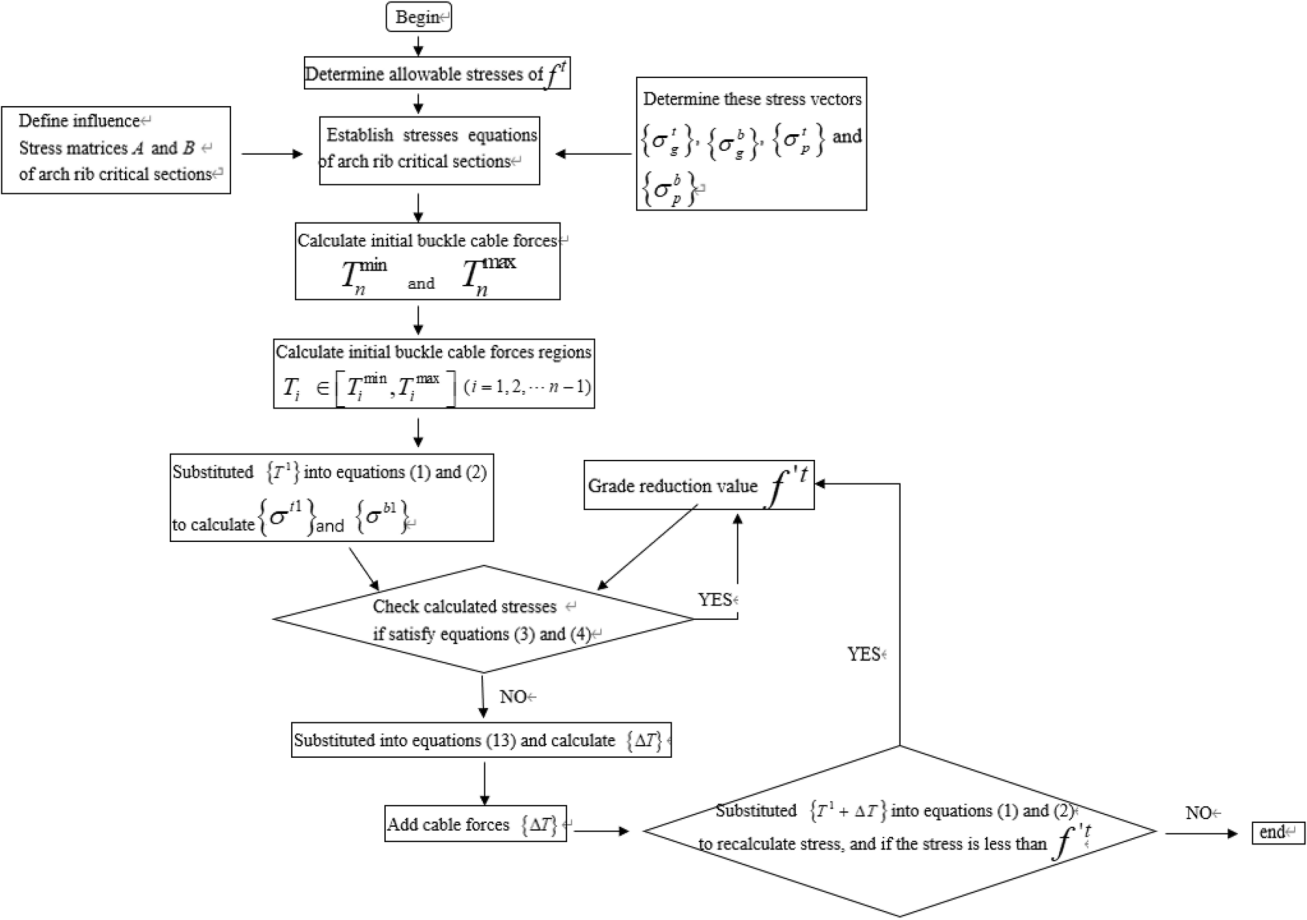
The calculation process of the initial buckle cable force.

If not, output optimized initial buckle cable force. Figure 2 shows the calculation process of the initial buckle cable force.

The anchor cable force can be calculated by the optimal initial buckle cable force. The main function of the anchor cable is to counteract the unbalanced horizontal force of the buckle cable on the tower. Therefore, the initial force of the anchor cable can be determined by the horizontal force equal to the initial force of the buckle cable.

## Engineering application

### Bridge description and finite element model

**Figure 3:**
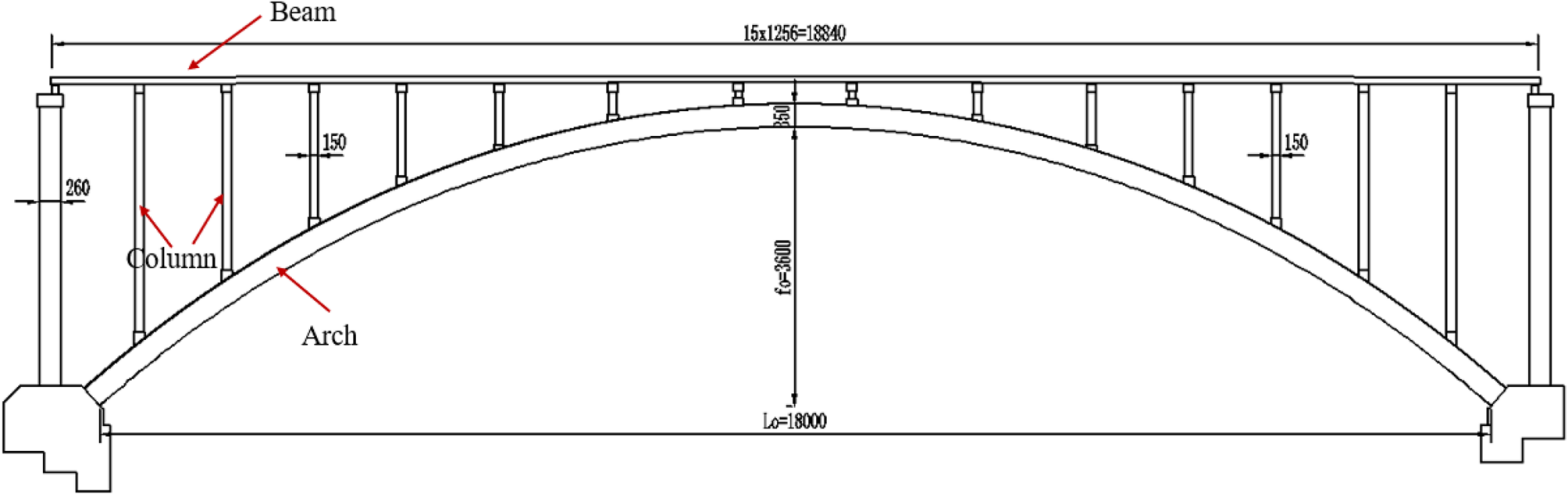
The overall drawing of Jiming Sansheng Bridge (unit: cm).

**Figure 4:**
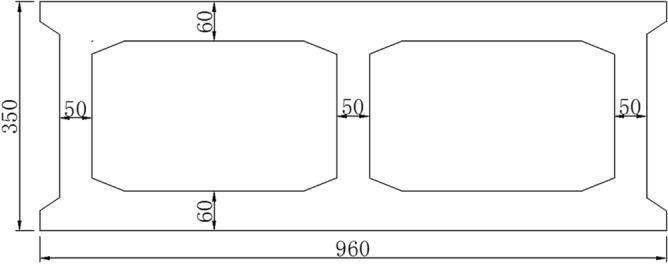
Arch foot section (unit: cm).

**Figure 5:**
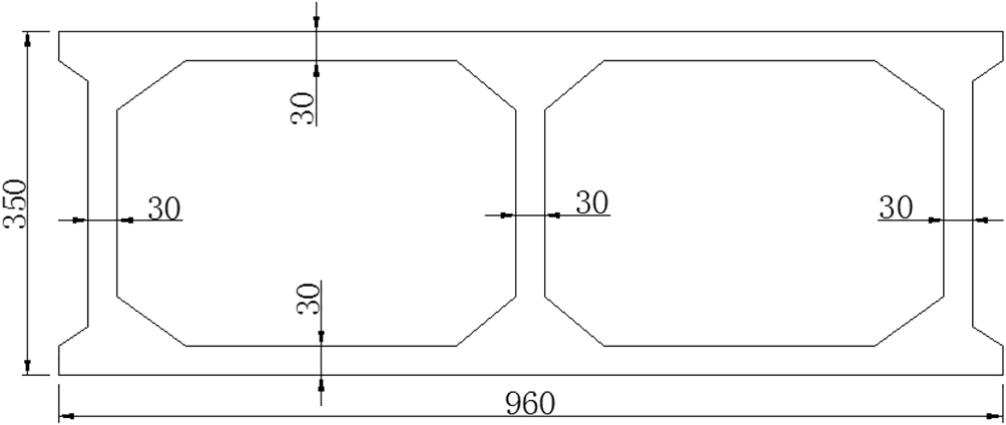
Arch rib standard section (unit: cm).

**Figure 6:**
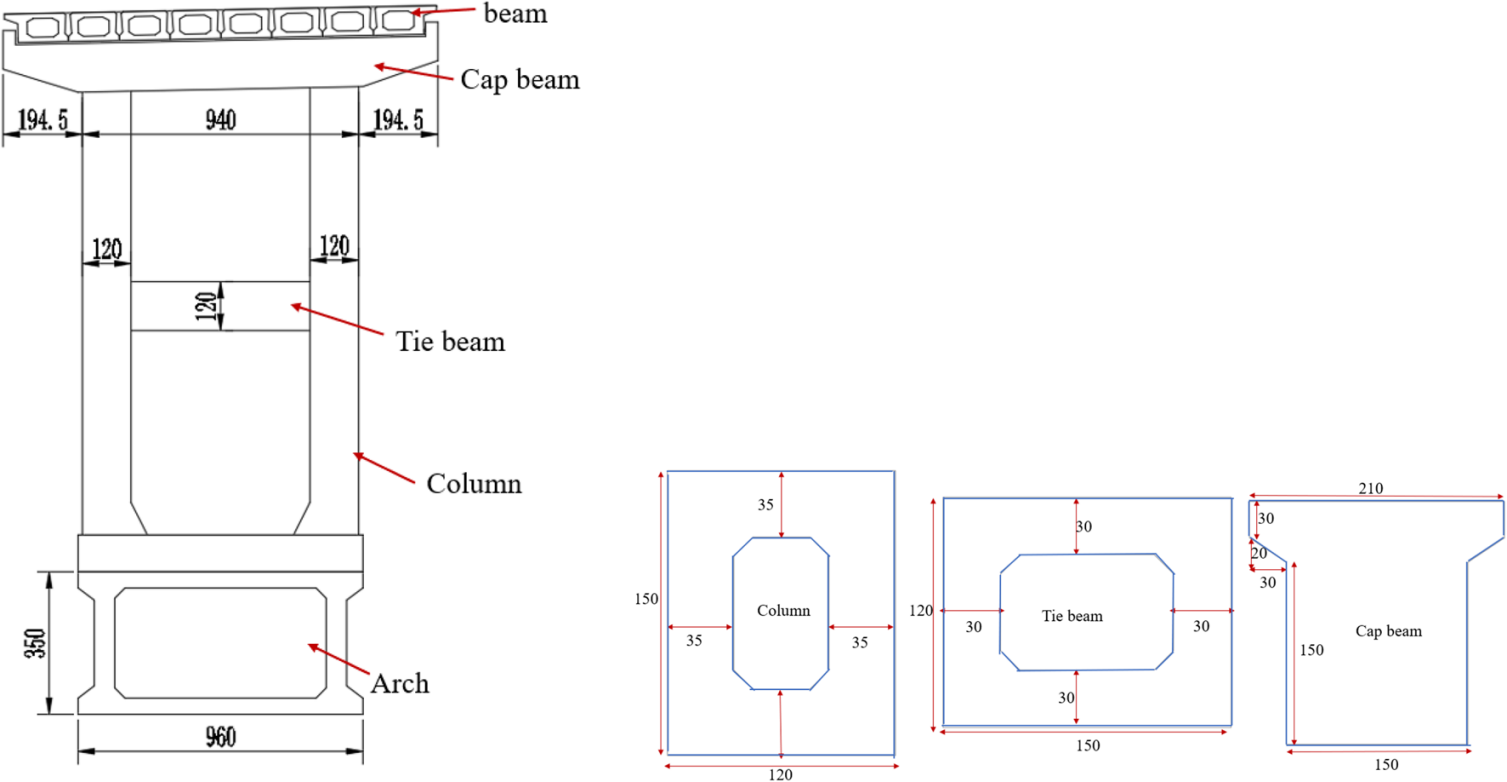
The column information diagram (unit: cm).

**Figure 7:**
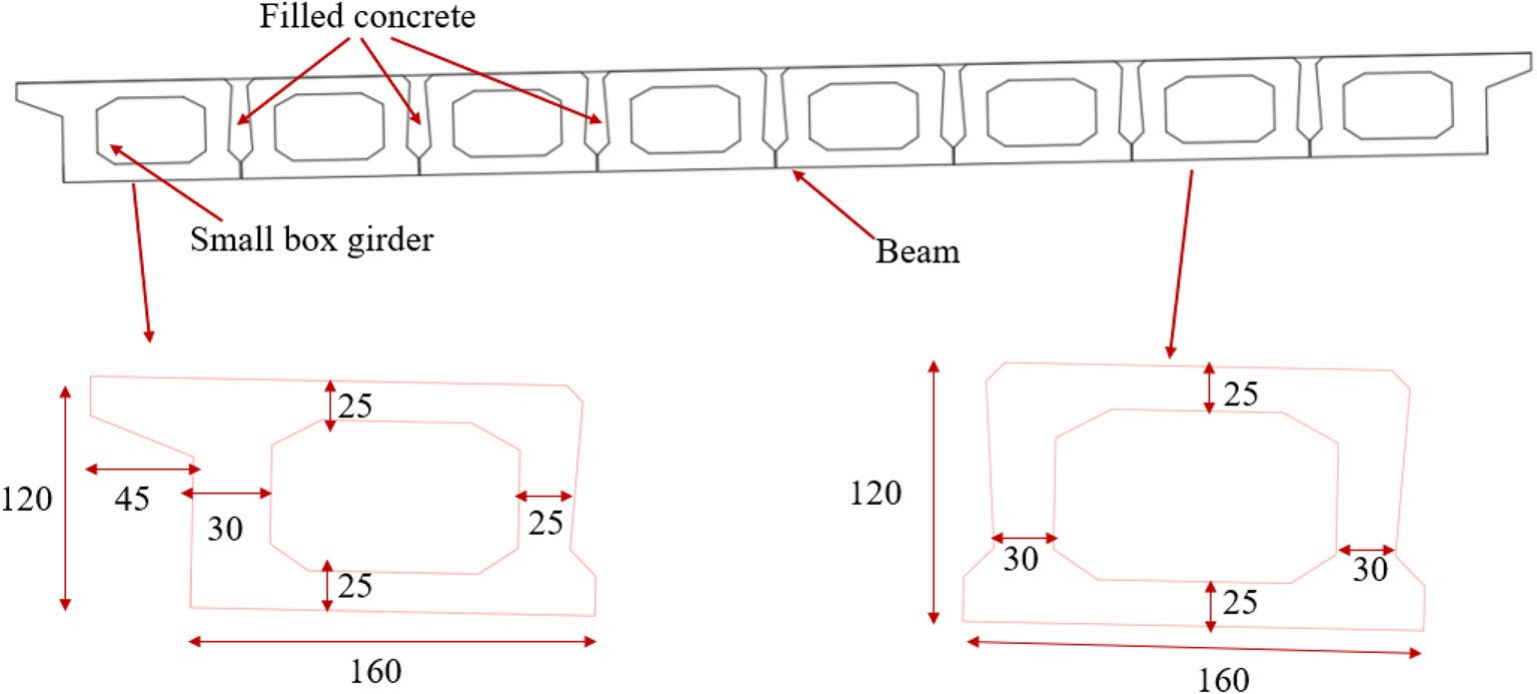
The main beam (unit: cm).

The Jiming Sansheng Bridge is a reinforced concrete arch bridge with a span of 180 m, located in Sichuan, China. The overall drawing of the Jiming Sansheng Bridge is shown in Fig. 3. The arch axis is a catenary. The rise height of the arch rib is 36 m, and the rise-span ratio is 1/5. Therefore, the arch axis coefficient is 1.988. The arch rib adopts box type section, and the section height is the same. The section height and width are 3.5 and 9.6 m respectively, as shown in Figs. 4 and 5. The thickness of the top plate, bottom plate and web of the arch rib standard section is 30 cm. The thickness of the top plate and bottom plate of the arch foot section is 60 cm, and the thickness of the web plate is 50 cm. The material of the arch rib is C50 concrete and the material of the buckle tower is Q235 steel. There are 14 columns in the whole bridge, the highest column is 30 m, and the columns are connected by tie beams. The top of the column is a cap beam, which supports the main beam. The column information diagram is shown in Fig. 6. The main beam is a simply supported beam with a span of 12.56 m, composed of multiple small box girders filled with concrete. The main beam is shown in Fig. 7.

**Figure 8:**
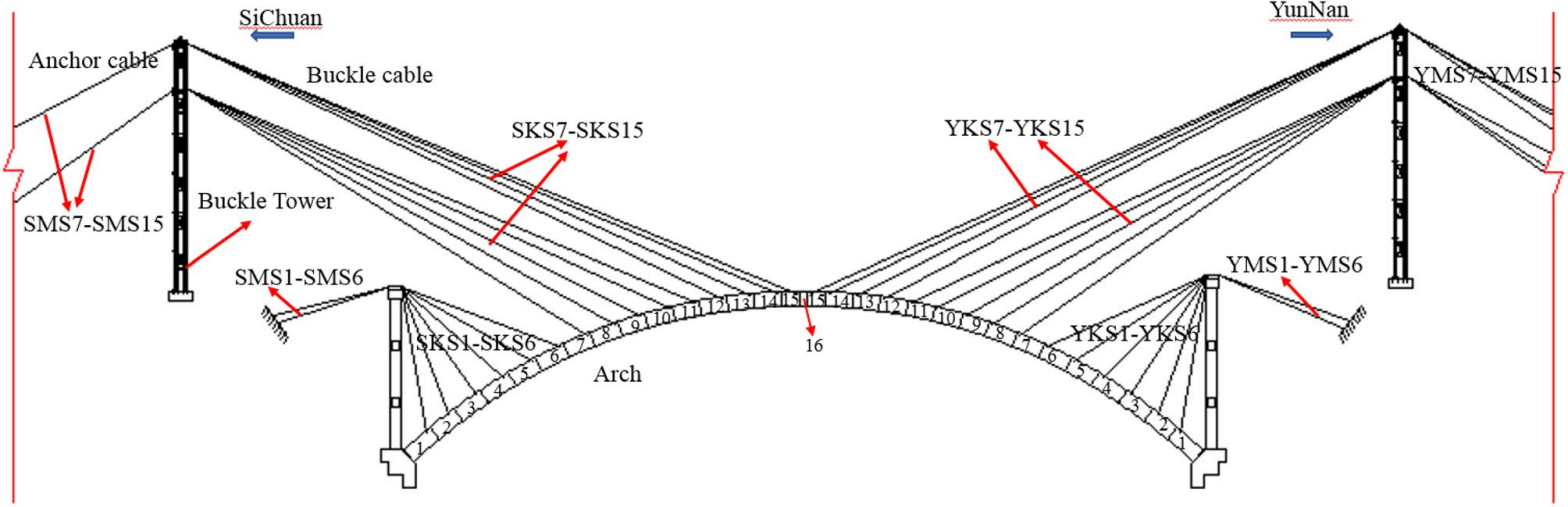
The construction layout of Jiming Sansheng Bridge.

**Figure 9:**
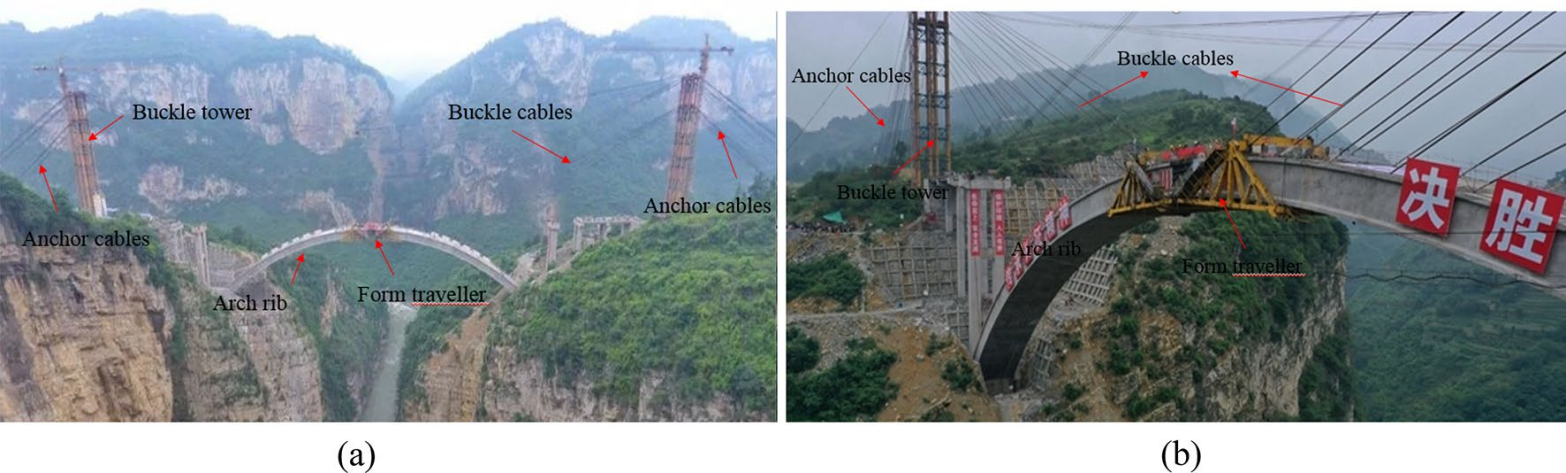
The site construction of Jiming Sansheng Bridge: (**a**) Panoramic view, (**b**) Partial view.

The arch rib was constructed by the cantilever casting method, with 31 segments. The construction layout of the Jiming Sansheng Bridge is shown in Fig. 8. The No.1 segment of the arch rib was constructed with support, and the No.2 ~ 15 segment was constructed with a hanging basket cantilever. The hanging basket was of triangle truss, with a weight of 80 t. The buckle and anchor cables are all composed of steel strands, and the number of steel strands was determined by the initial cable force. There were 15 pairs of buckle and anchor cables in the Sichuan and Yunnan banks. No.1 ~ 6 buckle and anchor cables were fixed on concrete piers, and the remaining cables were fixed on the buckle tower. The buckle cables on the Sichuan bank were named SKS1 to SKS15, and the anchor cables were named SMS1 to SMS15. Figure 9 is the site construction of the arch rib.

Midas/Civil is a finite element software that can simulate the three-dimensional stress of bridges. It can use truss element to simulate cable, beam element to simulate arch rib segment, concentrated force to simulate hanging basket, and can also simulate the gravity of arch rib. In addition, Midas/Civil can simulate the complex construction process, which can well meet the finite element analysis of cantilever pouring arch rib.

**Figure 10:**
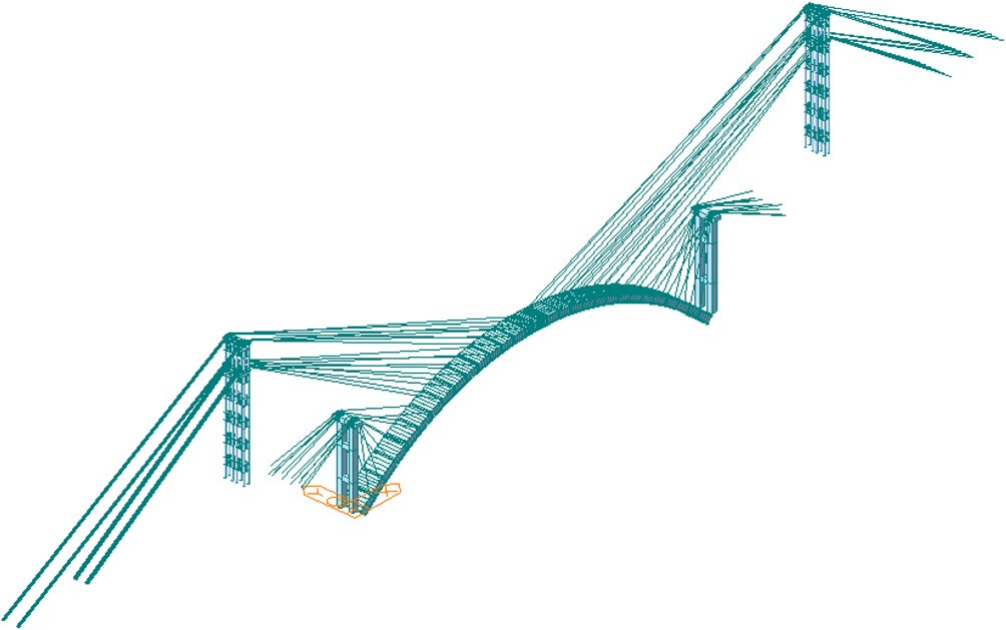
Finite element calculation model of Jiming Sansheng Bridge.

Midas/Civil was used to simulate the whole construction process of the arch rib of Jiming Sansheng Bridge, as shown in Fig. 10. The buckle and anchor cables were simulated by the truss element, and the Ernst formula was used to modify the elastic modulus. Other members were simulated by beam elements, such as the arch rib, pier and tower. The allowable tensile stress $f^{t}$ and compressive stress $f^{c}$ of concrete arch rib are 1.83 MPa and 22.4 MPa respectively (JTJ 2018). Fixed restraint was adopted at the consolidation end of the arch foot and anchor cable. The rigid connection was adopted for the connection of buckle cable and arch rib, and master–slave constraint was adopted for the connection of buckle cable, anchor cable, and buckle tower. The hanging basket is added to the arch rib in the form of node load.

**Figure 11:**
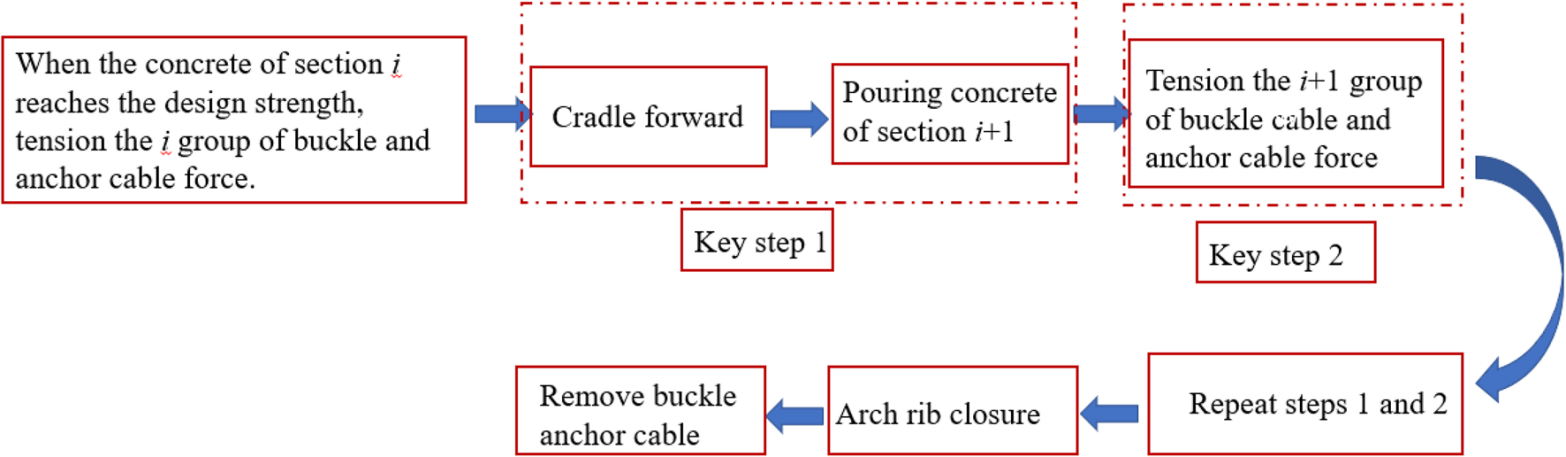
The simulation sequence of the construction phase.

**Figure 12:**
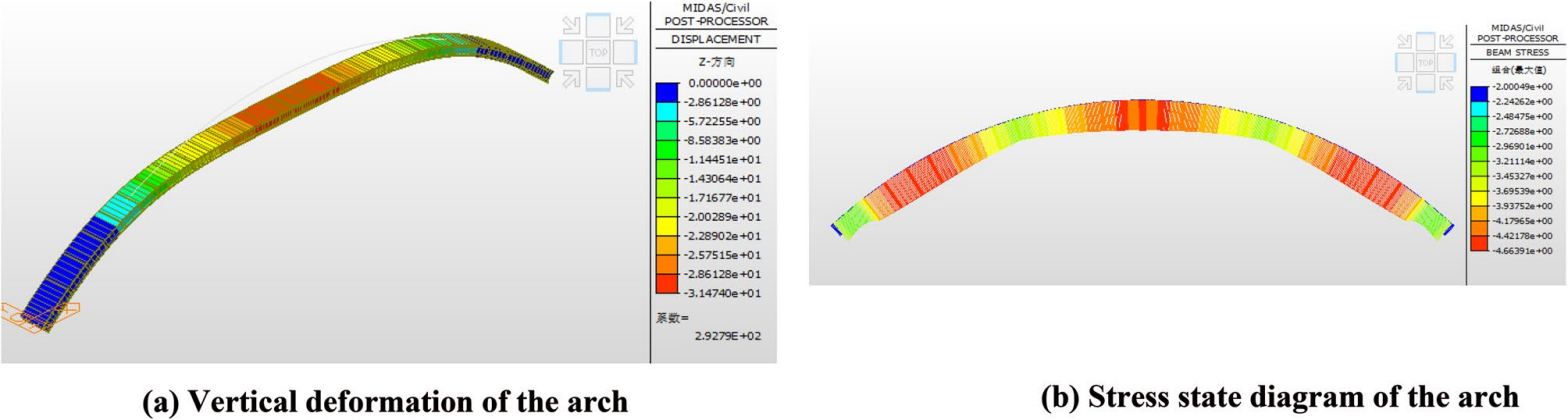
The stress and deformation of the arch after cable removal. (**a**) Vertical deformation of the arch. (**b**) Stress state diagram of the arch.

The simulation sequence of the construction phase is shown in Fig. 11 below. In key step 1, the upper edge of the arch rib section is tensioned, the lower edge of the section is compressed, and the arch produces downward deformation. In key step 2, the upper edge of the arch rib section is compressed and the lower edge of the section is tensioned due to the support of the cable, resulting in upward deformation of the arch. In key steps 1 and 2, the stress state of the arch rib is the same as that of the curved cantilever beam. However, after the arch rib is closed, the buckle and anchor cables are removed in sequence from the arch foot to the arch crown, and the arch rib bears pressure and produces downward deformation. The stress and deformation of the arch after cable removal are shown in Fig. 12.

### Construction monitoring

**Figure 13:**
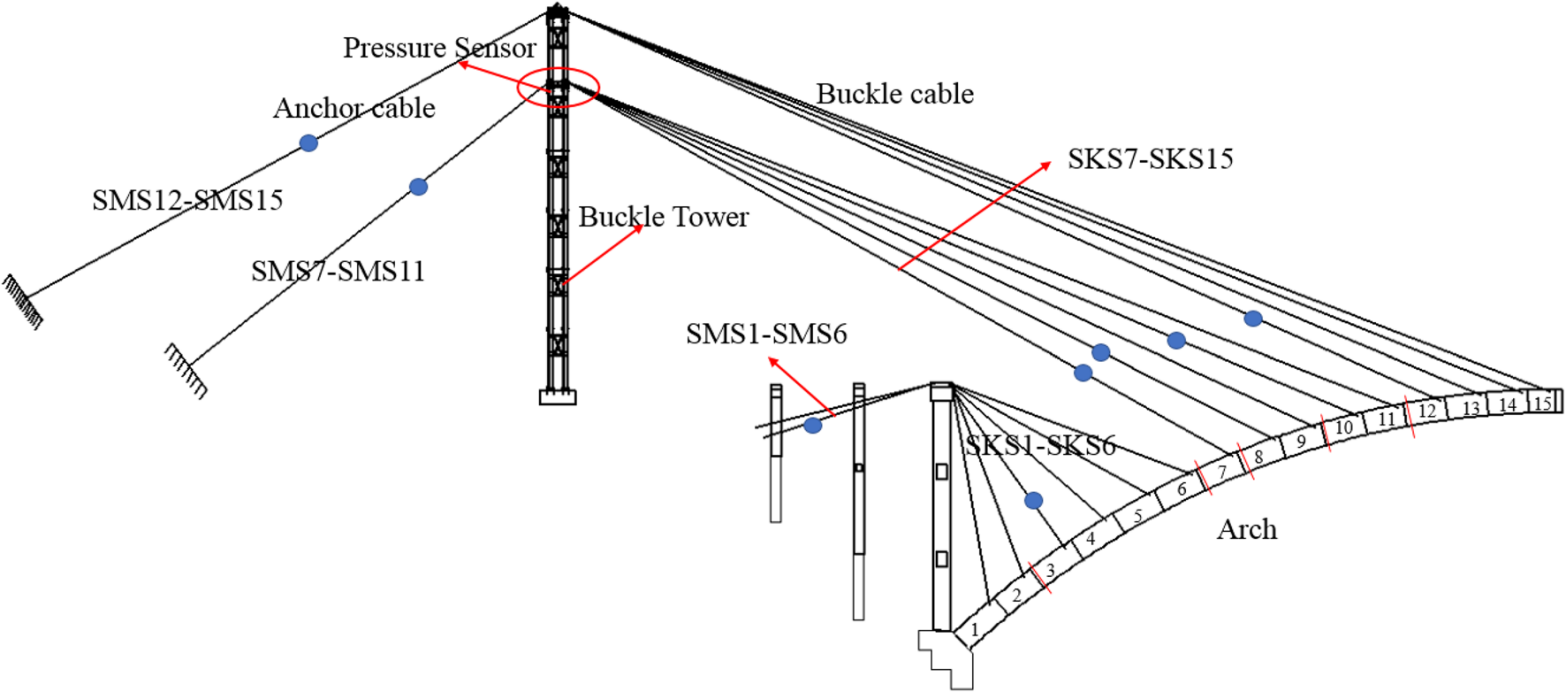
The layout of pressure sensors in Sichuan bank.

**Figure 14:**
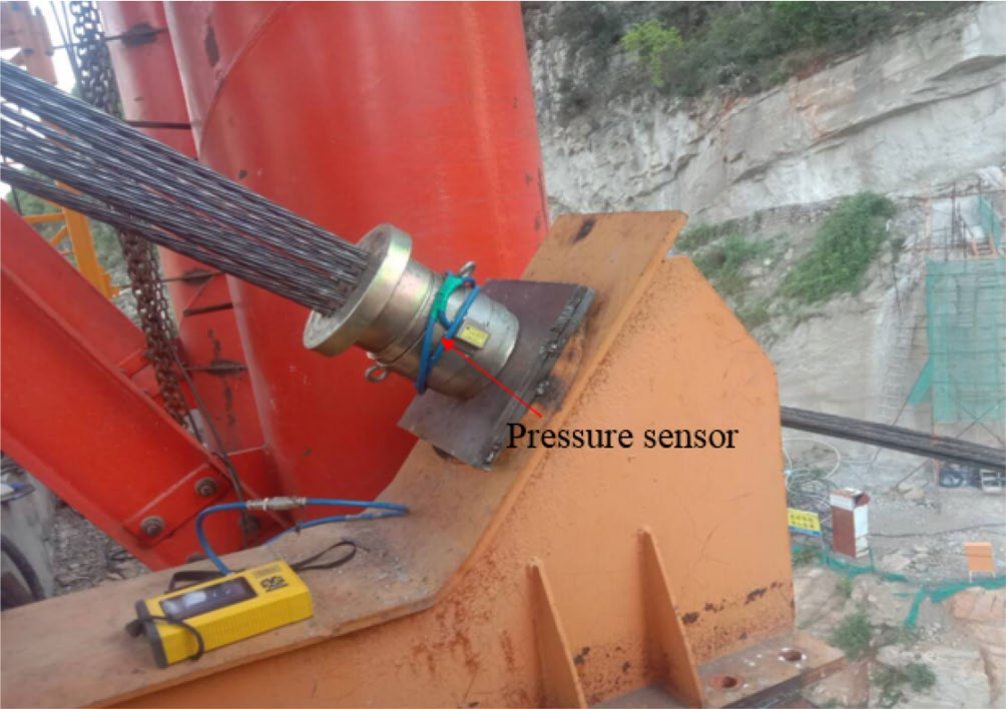
The physical figure of the pressure sensor.

The cable force affects the stress of the arch rib during construction. If the tensile stress is too large, it will affect the safety of the arch rib. Therefore, stress and cable force need to be closely monitored. Pressure sensors were fixed at the tension ends of buckle and anchor cables to measure their cable forces. The layout of pressure sensors in the Sichuan bank is shown in Fig. 13. Figure 14 is the physical figure of the pressure sensor.

**Figure 15:**
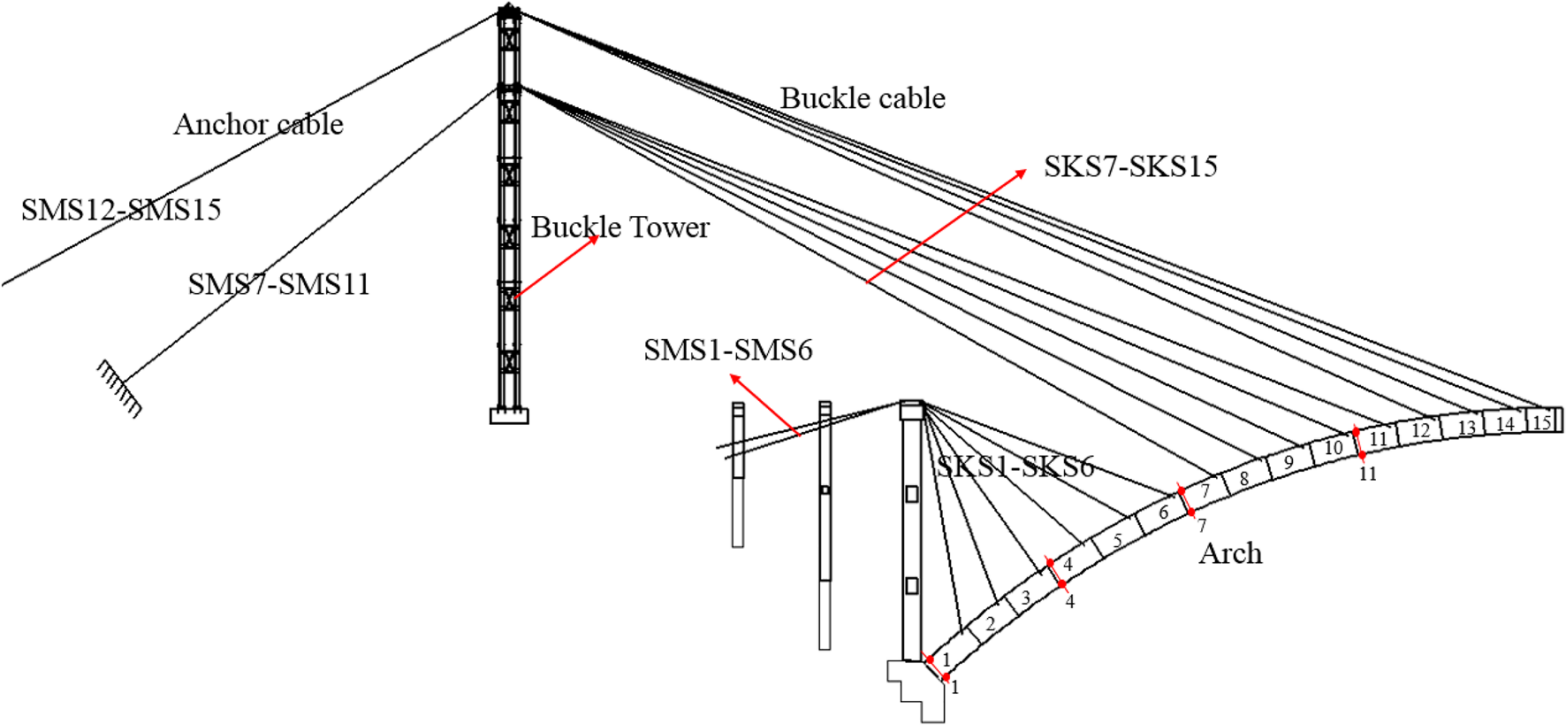
Strain monitoring sections.

**Figure 16:**
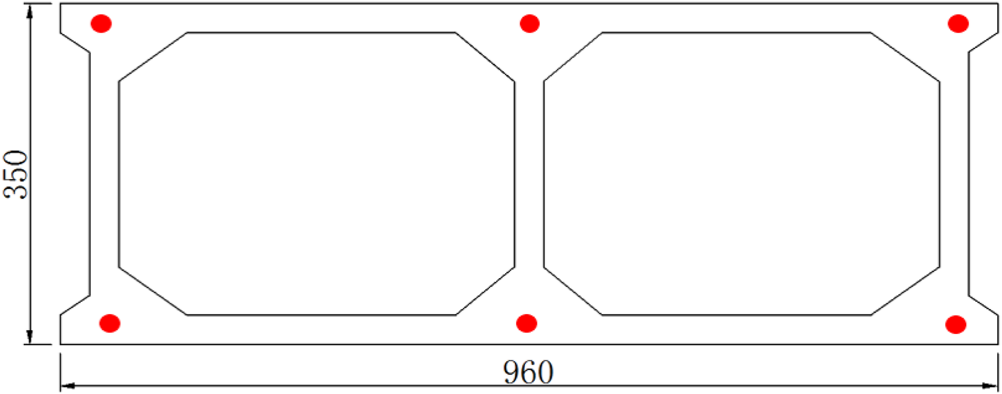
Arrangement of stress sensor in the arch rib section.

**Figure 17:**
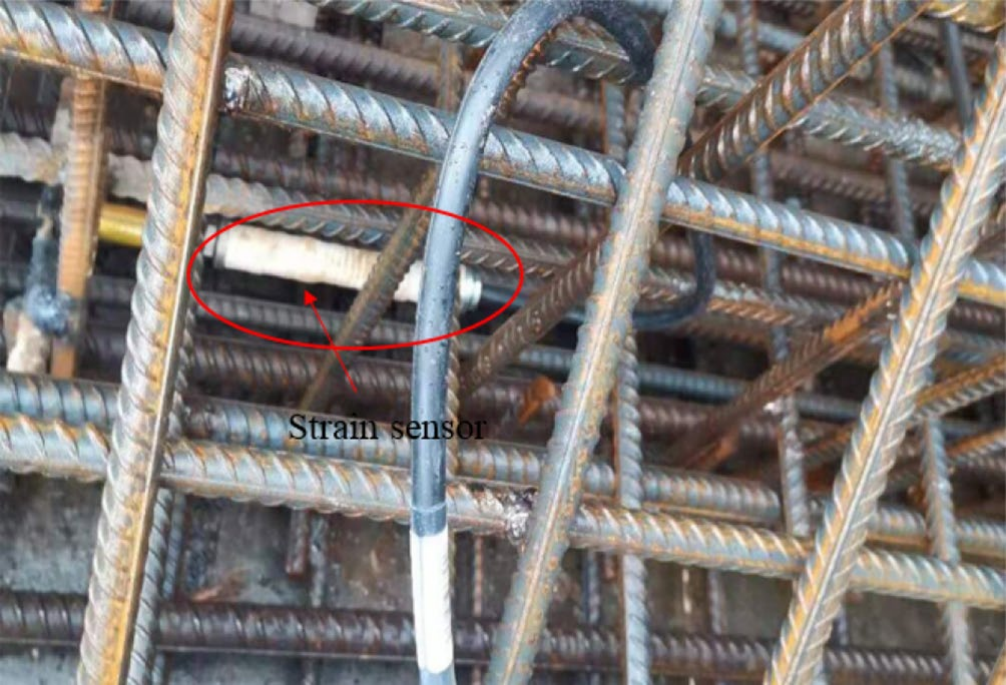
The scene of the concrete stress sensor.

The stress of arch rib concrete was measured by a vibrating wire sensor named JMZX-215AT. Stress sensors were installed at the key parts of the arch, such as the arch rib foot, the 4th segment, the 7th segment and the 11th segment, see Fig. 15. Six sensors were arranged in each section, as shown in Fig. 16. The scene of the concrete stress sensor is shown in Fig. 17.

### Engineering results and analysis

The arch rib of the Jiming Sansheng Bridge is symmetrical, and half of the arch rib has 15 key sections. The allowable tensile stress $f^{t}$ is 1.83 MPa. Through the finite element software, stress vectors ($\left\{ {\sigma_{g}^{t} } \right\}$, $\left\{ {\sigma_{g}^{b} } \right\}$, $\left\{ {\sigma_{p}^{t} } \right\}$ and $\left\{ {\sigma_{p}^{b} } \right\}$), as well as influence matrices A and B can be determined. Specifically, the arch rib dead weight, hanging basket load and cable force are set as separate load cases, which has the advantage that the action effects of different loads are separable. Then, the cable force feasible region of $T_{n}^{{}}$ can be determined according to Eqs. (9) and (10). The feasible region of all cable forces can be determined according to Eqs. (11) and (12).

**Figure 18:**
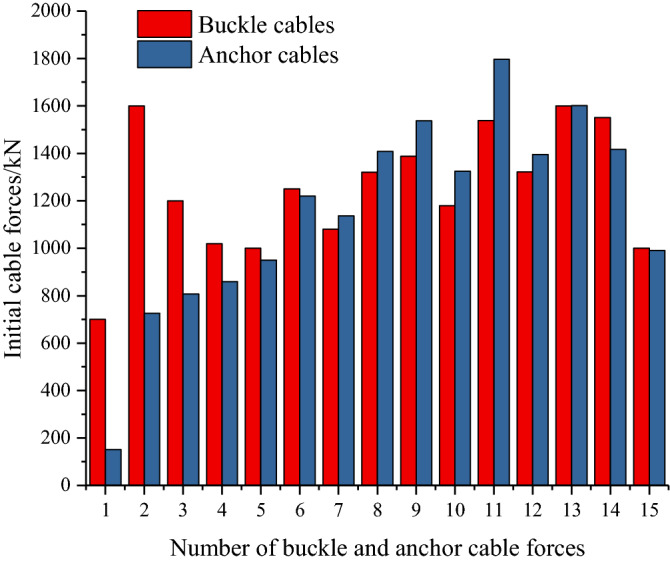
Initial cable forces.

This paper only lists the results of half span bridge. A group of buckle cable forces $\left\{ {T^{1} } \right\}$($\left\{ {T^{1} } \right\} = (T_{1}^{1} ,T_{2}^{1} ,T_{3}^{1} \ldots T_{n}^{1} )^{{\text{T}}}$) was selected from the feasible region of buckle cable forces. According to the calculation method and process of the optimal initial cable force, the optimal initial buckle cable forces and the corresponding allowable tensile stress $f^{^{\prime}t}$ (1.52 MPa) were obtained. The initial anchor cable forces were calculated according to mechanical balance principle. Figure 18 shows the final initial buckle cable forces and anchor cable forces of the Jiming Sansheng bridge. Among buckle cables, the initial cable force of buckle SKS2 is the largest, which is 1602 kN. The initial cable force of buckle SKS1 is the smallest, 701 kN. Among anchor cables, the initial cable force of anchor SMS11 is the largest, which is 1789 kN. The initial cable force of anchor SMS1 is the smallest, 151 kN. The large deviation of the anchor cable force of No. 1 and No. 2 is caused by the large horizontal inclination angle of the buckle cable and the small horizontal inclination angle of the anchor cable.

**Figure 19:**
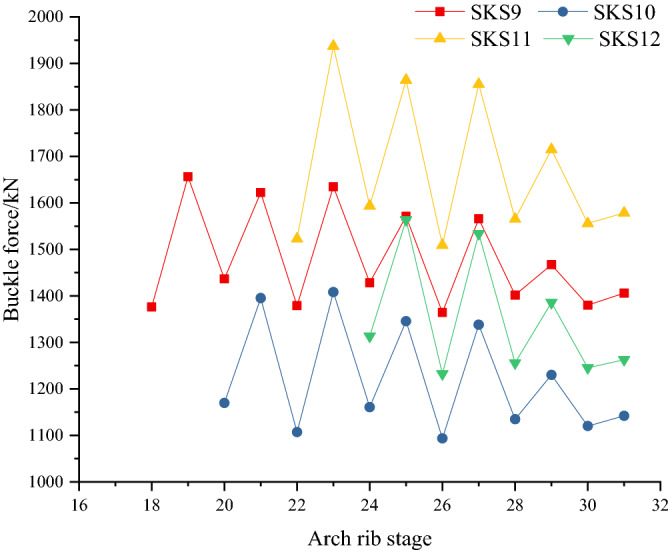
Variation curves of cable forces.

The change of cable forces during cantilever casting of the arch rib is shown in Fig. 19, which lists 9, 10, 11, and 12 buckle cables.

In Fig. 19, the change law of cable force of four buckles is basically the same, showing a fluctuating trend. The buckle cable force has a great change during the concrete casting stage and the tensioning stage of the buckle cable. For example, the cable force SKS9 is 1376.2 kN in the tensioning stage, and it becomes 1656.5 kN when pouring the arch rib No.10 segment concrete, subsequently, 1436.7kN in the tensioning stage of buckle cable SKS10. In addition, the fluctuation range of cable forces gradually decreases with the increase of the construction stage. The above phenomenon is that the buckle cable has a supporting effect on the arch rib. The closer the pouring concrete is to the buckle cable, the more the weight of the concrete is transferred to the buckle cable, resulting in an obvious increase in the buckle cable force. After the later cable is tensioned, it bears the gravity of the newly poured concrete, reducting of the previous cable force. With the support of more new buckles, the impact of concrete poured later on the previous buckles is gradually reduced. It also shows that the role of the cable buckle is very important in the process of arch rib construction, and the cable force calculation method in this paper is very meaningful. Moreover, the change law of buckle cables is the same in the same construction stage, showing that the cable forces increase or decrease simultaneously. This shows that the buckle cables can give full play to the role, in line with the principle of uniform distribution of buckle cable forces.

**Figure 20:**
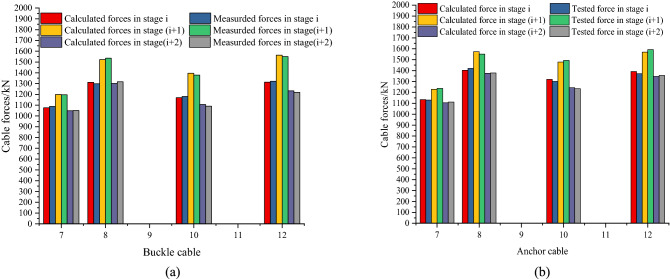
Comparison of cable forces between calculation and measurement in different construction stages: (**a**) Buckle cable forces in different construction stages, (**b**) Anchor cable forces in different construction stages.

Figure 20 shows the comparison of cable forces between calculation and measurement in different stages. Only the forces of No.7, No.8, No.10, and No.12 buckle and anchor cables are listed. Stage *i* is to tension the buckle and anchor cables of arch rib segment *i*. The stage (*i* + 1) represents the concreting of the arch rib segment (*i* + 1). The stage (*i* + 2) is to tension the buckle and anchor cables of the arch rib segment (*i* + 2).

In Fig. 20, the cable forces after tension increase first and then decrease in the next two stages.

For example, the calculated and measured values of buckle cable SKS7 in the tension stage are 1075 kN and 1089 kN respectively. Stage (*i* + 1) increased to 1198 kN and 1183 kN respectively. Stage (*i* + 2) is reduced to 1047 and 1051 kN respectively. Moreover, the difference between calculated and measured cable forces is very small in different construction stages, and the maximum deviation is less than 2%. The cable force changes obviously in these three construction stages. Through the comparison of three consecutive construction stages, it can be seen that the cable force calculation method in this paper is effective and can provide a reference for the cable force calculation of a cantilever concrete arch bridge.

**Figure 21:**
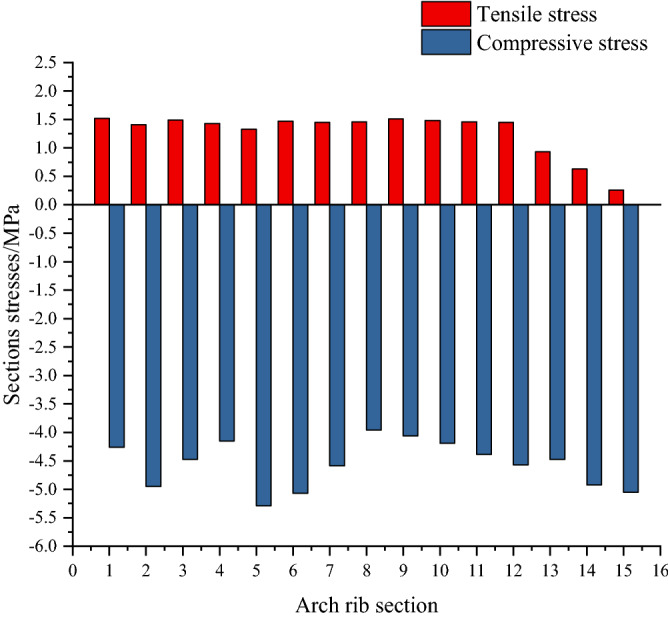
Stress ranges of the top edge of arch rib key sections.

**Figure 22:**
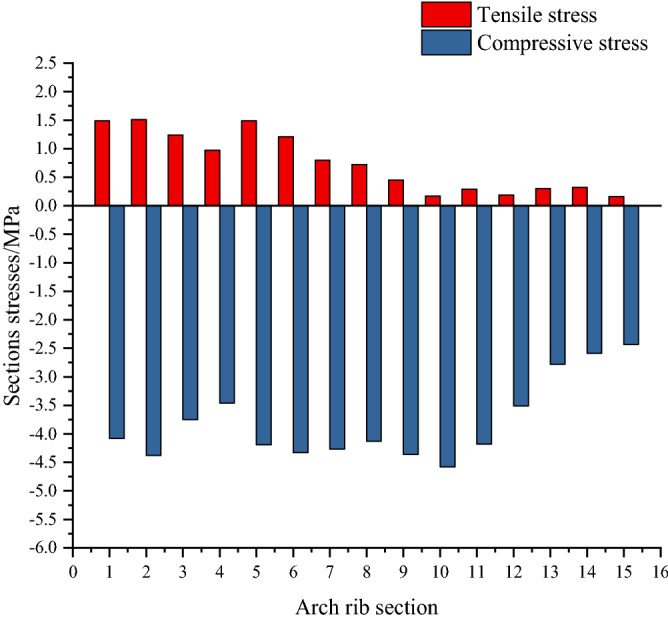
Stress ranges of the lower edge of arch rib key sections.

Figures 21 and 22 show stresses on the upper and lower edges of the key section of the arch rib. In Fig. 17, the maximum tensile stress on the upper edge of the key section of the arch rib is 1.52 MPa, which is the No.1 section (the arch foot section). The maximum compressive stress is − 5.35 MPa, the No.2 section. In Fig. 18, the maximum tensile stress at the lower edge of the key section of the arch rib is 1.50 MPa, which is the No.5 section. The maximum compressive stress is − 4.58 MPa, the No. 10 section. Therefore, the allowable tensile and compressive stresses in the specification are 1.83 MPa and − 22.4 MPa, respectively, and the arch rib stresses meet the requirements of the specification.

The allowable compressive stress (− 22.4 MPa) in the specification is far less than the maximum compressive stress (− 5.35 MPa) of the key section of the arch rib. This shows that it is feasible to calculate the feasible region of cable force based on the stress balance method only considering the tensile stress of the key section. The allowable compressive stress specified in the code is used to calculate the bearing capacity of the bridge, and many extremely unfavorable loads are considered. Therefore, the required compressive stress in the code is too conservative, which also shows that the method proposed in this paper to consider only the tensile stress of arch rib is scientific.

**Figure 23:**
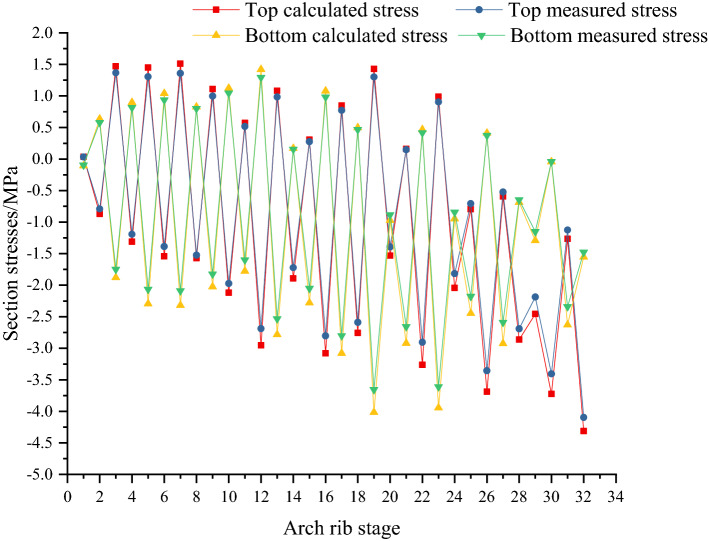
Calculated and measured stresses of key section No. 1 (arch foot) in the process of arch rib construction.

**Figure 24:**
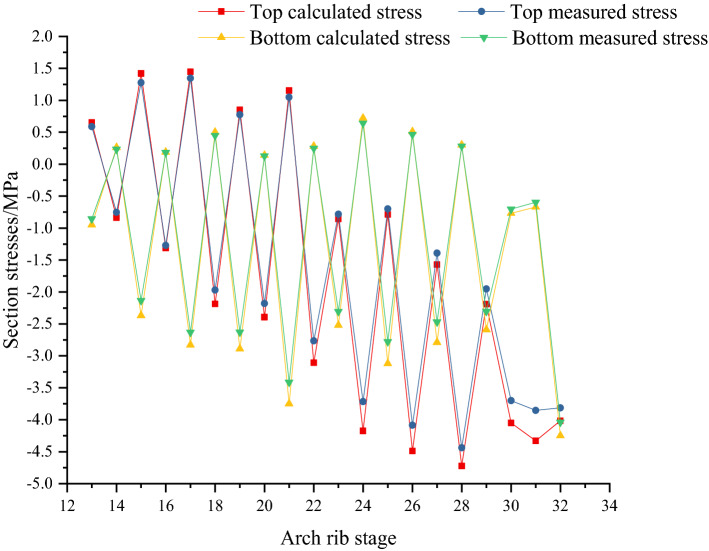
Calculated and measured stresses of key section No. 7 in the process of arch rib construction.

Figures 23 and 24 show the calculated and measured stresses of key sections in different construction stages. Stresses of sections 1 (arch foot) and 7 of the arch rib are shown in Figs. 23 and 24 respectively. In Figs. 23 and 24, with the progress of the construction stage, the stress curve shape of the upper and lower edges of key sections presents the cycle of crossing and separating, and the stress finally changes into compressive stress. The stress of the upper and lower edge of the section shows a downward trend. The tensile stresses at the upper edge of arch rib sections increase at the concrete cantilever casting stage, and then change to compressive stress at the cable tensioning stage. The changing trend of stress at the lower edge is opposite to that at the section's upper edge. After pouring the concrete of the arch rib segment, the arch rib will move downward, resulting in tensile stress at the section's upper edge. If the cable force is too small, the tensile stress at the upper edge of the arch rib section will continue to increase. If the cable force of the cable is too large, the lower edge of the arch rib may produce tensile stress. Therefore, the initial tension of the cable is very important. In addition, the calculated stresses are very consistent with the measured stresses, and the measured stresses are less than the calculated stresses. The maximum deviation between measured and calculated stresses is 11.5%, less than 12%. The main reason for the deviation between measured stress and calculated stress is that the effect of concrete shrinkage and creep is not considered. Concrete shrinkage and creep are complex and nonlinear. For concrete arch bridges, the effect of shrinkage and creep is similar to that of reducing the temperature, which causes compressive stress in arch ribs, so it is not considered in this paper.

## Conclusions

Taking Jiming Sansheng bridge as the engineering background, this paper studies the calculation method of initial buckle cable force. This paper put forward one method to calculate the feasible region of the initial cable force based on the allowable tensile stress of the arch rib. The stress balance equation considering only tensile stress was derived for the first time, and the feasible region of the initial cable force was calculated by the allowable tensile stress of the arch rib, which improved the original stress balance method. Furthermore, the allowable tensile stress was reduced step by step using the influence matrix to optimize the initial force. The practical engineering results show that the calculation method put forward in this paper is feasible. Furthermore, the research results ensure the smooth completion of the Jiming Sansheng bridge.

1. Through the engineering example, the change law of cable force of four buckles is basically the same, showing a fluctuating trend. The buckle cable force has a great change during the concrete casting stage and the tensioning stage of the buckle cable. The tensile stresses at the upper edge of arch rib sections increase at the concrete cantilever casting stage, and then change to compressive stress at the cable tensioning stage. The changing trend of stress at the lower edge is opposite to that at the section's upper edge.
2. The difference between the calculated and measured cable forces is very small in different construction stages, and the maximum deviation is less than 2%. Moreover, the calculated stresses are very consistent with the measured stresses, and the measured stresses are less than the calculated stresses. The maximum deviation between measured and calculated stresses is 11.5%, less than 12%. The practical engineering results show that optimizing the initial buckle force is feasible by reducing the allowable tensile stress.
3. In the process of arch rib construction, the buckle and anchor cables are only tensioned once, which greatly shortens the construction time and increases the safety of the cantilever casting arch rib.

The method and experience of this paper can provide a reference for the arch bridge constructed by cantilever casting. However, it should be pointed out that the influence of extreme adverse loads such as wind load and earthquake have not been considered in the cable force calculation method in this paper.

## Data Availability

All data generated or analysed during this study are included in this published article.
